# Statue of Vesalius

**Published:** 1844-05-18

**Authors:** 


#### STATUE OF VESALIUS.

A colossal statue of bronze, 11 feet high, will be erected to André Vesalius, in one of the public squares of Brussels, his native town, on the 18th July 1845.

